# Improving science teachers’ nature of science views through an innovative continuing professional development program

**DOI:** 10.1186/s40594-018-0125-4

**Published:** 2018-07-17

**Authors:** Eda Erdas Kartal, William W. Cobern, Nihal Dogan, Serhat Irez, Gultekin Cakmakci, Yalcin Yalaki

**Affiliations:** 1 Department of Educational Sciences, Kastamonu University, Kuzeykent, 37150 Kastamonu, Turkey; 2 Mallinson Institute for Science Education, Western Michigan University, 1903 W. Michigan Avenue, Kalamazoo, MI 49008 USA; 3 Department of Elementary Science Education, Abant Izzet Baysal University, Golkoy, 14280 Bolu, Turkey; 4 Department of Biology Education, Marmara University, Goztepe, 34722 Istanbul, Turkey; 5 Department of Elementary Education, Hacettepe University, Beykent, 06800 Ankara, Turkey; 6 Department of Elementary Science Education, Hacettepe University, Beykent, 06800 Ankara, Turkey; 7 Department of Educational Science, Education Faculty, Kastamonu University, City Center, 37200 Kastamonu, Turkey

**Keywords:** Nature of science, Professional development, Teacher education, Science education

## Abstract

**Background:**

This study describes how teachers’ nature of science (NOS) views changed throughout an innovative Continuing Professional Development (CPD) program that provided sustained support throughout the process in a collaborative and reflective environment and activities that are consistent with the current curriculum and NOS tenets integrated within. Eighteen in-service science teachers enrolled in a yearlong nature of science, Continuing Professional Development (NOS-CPD) program. Data were collected by pre/post-interviews using the Views of Nature of Science-Form C (VNOS-C) questionnaire, and a post-interview using an open-ended questionnaire developed by researchers to uncover teacher reactions to the NOS-CPD program.

**Results:**

The results indicated that the NOS-CPD program improved the teachers’ NOS views more effectively than previously reported short-term teacher development programs, and thus, the findings should be useful for future studies in support of the professional development of teachers.

**Conclusions:**

The article concludes with practical advice for implementing NOS-focused, in-service teacher development programs.

*The transformational change agent says: “Here is the standard, which I know is impossible, so let’s stand together and learn our way into a higher level of performance.”*

*Robert Quinn (2000, p.164)*

## Background

Equipping individuals with adequate knowledge and understanding of science and technology has become one of the main goals of national education programs (e.g., Ministry of National Education (MONE Turkey) 2004, 2013; Next Generation Science Standards in the USA (NGSS Lead States) 2013). Calls for scientific literacy echo across many countries. These national science education programs include goals for understanding the nature of science (NOS) as an important component of scientific literacy. Although there is no NOS consensus definition (Cobern and Loving 2001; Lederman 1992) much of the science education community, nevertheless, agrees that NOS should be highlighted in the curriculum and taught to students (Lederman 1992). And, there are broadly accepted models of the NOS. Unfortunately, studies show that Turkish students often have inadequate views and misconceptions about NOS (Lederman and Lederman 2014; Ozer 2014; Park et al. 2013).

Numerous studies have tested methods for improving students’ NOS views. Although these studies show that NOS instruction can be made more effective, the studies indicate that there is room for still the improvement of students’ understanding of NOS. Moreover, NOS studies suggest that some science teachers have naive conceptions about NOS and numerous misconceptions (Akerson et al. 2009; Dogan and Abd-El-Khalick 2008; Guerra-Ramos et al. 2010). Teachers need to have informed NOS views since they cannot help their students understand what they themselves do not understand (Capps et al. 2012; Loucks-Horsley and Matsumoto 1999).

Thus, effective professional development opportunities are important for helping teachers to improve their understanding of NOS, and research with teachers shows that professional development programs can improve teachers’ NOS views (Akerson et al. 2009; Ozer 2014). The literature indicates that effective professional development programs have the following features:

### Based on teacher needs

Designed to fit personal needs of the participating teachers (Gess-Newsome 2001).

### Coherency with other reform initiatives

Stresses reform-oriented practices such as teacher mentoring or coaching, participating in a committee or study group (Garet et al. 2001) with a focus on curriculum linked activities rather than general pedagogical strategies (Cohen and Hill 2001).

*High-quality instruction:* Explicitly designed to improve teachers’ content knowledge and practices (Bertram and Loughran 2012; Posnanski 2010).

### Active engagements of teachers

Based on the principles of active learning (Boone and Kahle 1998; Marek and Methven 1991).

### Enhancement of both content knowledge and pedagogical content knowledge

Stresses the importance of both content and pedagogical knowledge (Shulman 1987).

### Provision of sufficient time and other resources

Provides sufficient time in a well-organized, carefully structured, and purposefully directed environment consistent with the curricula and provides relevant resources and materials.

*Sustained support:* Provides continuing support that helps them overcome these challenges (Capps et al. 2012).

### Ensuring collaboration

Provides opportunities for teacher collaboration (Putnam and Borko 1997).

### Provision opportunity for reflection and giving feedback throughout the professional development program process

Provides opportunities for teacher reflection on what they are learning and how they will apply what they learned (Loucks-Horsley and Matsumoto 1999). Provides feedback to teachers using these reflective comments made by teachers, and so, these reflective comments can be a valuable tool for teacher learning and teacher change (Capps and Crawford 2013).

### Provision of local support

Develop local support for teachers when they return to their classrooms (Kwakman 2003; Penuel et al. 2007).

Where professional development programs often fail is with the “provision of sufficient time” and “sustained support.” Researches show that long-term professional development programs are more effective than short-term programs (Dass and Yager 2009) because learning to teach and fundamental change in practice is not easy and takes time (Guskey and Yoon 2009). Too often professional development does not follow teachers back to the classroom where teachers may face some challenges and problems while translating their new understanding into performance. The limited time in these professional development programs does not allow this. Effective professional development requires supporting teachers in the transfer of what they learned into practice (Gess-Newsome 2001; Ozer 2014).

The above comments are about professional development in general. Our specific interest is professional development with respect to the NOS. Given the importance of sustained professional development support as discussed above, our study planned a CPD program that provides teachers with such support. As professional development in support of teachers’ understanding of NOS, our approach followed research findings in two important areas:

1. Researches have demonstrated that explicit-reflective instruction in teaching NOS is typically more effective than implicit instruction (Abd-El-Khalick and Lederman 2000; Khishfe and Abd-El-Khalick 2002).
2. Researches have also demonstrated that NOS instruction can be more effective when *context-specific* activities are used rather than *generic* activities (Cakmakci 2012; Sadler et al. 2010).

In addition to observing the above research-based, professional development practices, we innovated by including formative assessment and discourse analysis within our NOS-CPD program. Researches have shown that using formative assessment rather than summative assessment can improve learning (Bennett 2011); summative assessment often comes too late to be much help (Guskey 2000). On the other hand, given that teachers use a variety of communication approaches and patterns of discourse in the classroom that impact student learning (Kaya et al. 2016; Mortimer and Scott 2003; Sinclair and Coulthard 1975), researches indicate that teacher-student communication in the classroom needs to be examined with regard to NOS issues (Herman et al. 2013). It is thought that teacher awareness of their NOS discourse patterns and communication approaches can be improved by analyzing classroom discourses. For these reasons, it has been decided to use these formative assessment and discourse analysis within the teaching of the NOS in our NOS-CPD program.

#### Aim of the study

This study evaluated the effectiveness of an innovative NOS-CPD program with specific attention paid to how teachers’ NOS views change throughout the sustained, CPD program. The main research question is: In response to an innovative NOS-CPD program that provided sustained support, to what extent and in what ways do teachers’ NOS views change? The subquestion then is: To what extent do these changes show improvement over short-term PD programs?

#### The NOS-CPD program innovation

**Fig. 1:**
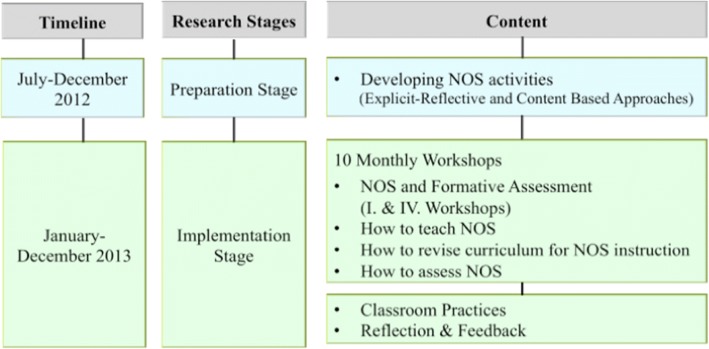
Process of the research

This paper reports the findings of our study on NOS-CPD program effectiveness. The NOS-CPD program was part of a large-scale Turkish teacher professional development research project intended to improve middle school in-service science teachers’ professional competences about NOS and consisted of a preparation stage and an implementation stage (see Fig. 1).

**Fig. 2:**
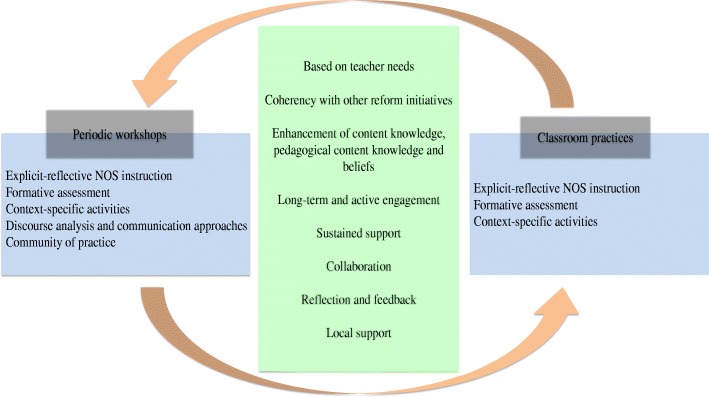
Model of the innovative NOS-CPD program

The NOS-CPD program consisted of NOS activities, with eight NOS themes emphasized in the activities: empirical NOS, scientific method, tentative nature of science, the nature of scientific theories and laws, inference and theoretical entities in science, the subjective and theory-laden NOS, the social and cultural embeddedness of science, and imagination and creativity in science. The themes were derived from: the general thematic structure of the VNOS-C (Lederman et al. 2002), the characteristics of NOS intended to be developed in this project, and the analytical frameworks used in several researches examining the understanding of various groups (e.g., students, teachers, scientists) about NOS (e.g., Irez 2006). The NOS-CPD program model is as follows as shown in Fig. 2.

Participating teachers in the NOS-CPD program attended 10, monthly workshops, each consisting of 8 h, over the course of two semesters. The teachers were introduced in a collaborative and reflective environment to various NOS aspects and ways of using explicit instruction and formative assessment in their NOS teaching. They were also introduced to different patterns of discourse and communication approaches by analyzing video recordings in the classroom. The teachers were also provided with opportunities to develop and use various context-specific NOS activities in their own classrooms during the study. During the workshops, context-specific NOS activities were introduced to the teachers and the teachers’ opinions about the activities were taken. Teachers were asked to apply the shared activities in their classes; the next workshop allowed them to reflect on their experiences and thoughts on their practice. Activities were reorganized according to the views and suggestions from the teachers. During this process, the teachers along with the researchers collaboratively produced 57 NOS activities all meeting the project criteria. The activities are available at Dogan et al. (2016).

Each of the activities consists of four sections: introduction, implementation, guidance for classroom discussion, and formative assessment. The activity introduction provides information on the subject matter, the purpose of the activity, and specifies the NOS themes taught by the activity, and what questions the students should be able to answer after the implementation. The implementation section provides guidelines just on how to implement the activity, including points to be emphasized during the implementation of the activity. The guidance for classroom discussion section provides teachers with instructional tips on the explicit-reflective teaching of NOS. Finally, in the formative assessment section of the activity, there were sample questions that will help the teacher to formatively assess students’ NOS achievement.

## Methods

### Participants

Eighteen (11 female, 7 male) in-service middle school science teachers (teaching grades 5 through 8) volunteered to participate in this study. These teachers worked in 15 different schools in Turkey. They regularly attended project meetings and fulfilled all participation requirements. Thirteen of 18 (72.2%) participating teachers had previously taken a short-term course or training about the history of science and philosophy of science or NOS.

### Data collection and analyses

The data were collected through interviews and analyzed using content analysis. Participant’s NOS understandings were assessed through face-to-face interviews at the beginning and end of the second stage. These interviews were semi-structured based on VNOS-C questions as developed by Abd-El-Khalick (1998). Although Abd-El-Khalick developed the original questionnaire as a paper-pencil instrument, the questions have been found appropriate for use in interviews (Irez 2006).

Analyses of the interviews were carried out in several steps. First, interviews were transcribed. Second, these transcripts were transferred to a qualitative data analysis program. Thirdly, teachers’ statements were grouped regarding NOS themes. At the 4th stage of the analysis, teachers’ statements about related themes were classified as *naive*, *eclectic*, and *informed.* Table 1 provides the “operational definitions” for the categories of naive, eclectic, and informed.

**Table 1:** “Operational definitions” for naive, eclectic, and informed

Category	View
Naive	Insufficient views about relevant theme of NOS
Eclectic	Inconsistent and often contradictory views about relevant theme of NOS
Informed	Consistent views with the contemporary approaches about relevant theme of NOS

A rubric was used that developed by Irez (2004), defining each of these categories for each theme, to aid the classifying procedure (Table 2). In this analysis, insufficient views about relevant theme of NOS were labeled naive, views characterized by inconsistent and often conflicting statements about the NOS were labeled eclectic, and consistent views with the contemporary approaches about relevant theme of NOS were labeled informed (Irez 2004; Koulaidis and Ogborn 1988). Before classifying all teacher statements according to themes, inter-rater reliability was checked. Participant transcripts were given to two raters for independent classification. Inter-rater reliability was found to be 82%. Differences were reconciled through discussion between the raters, then all teacher statements classified. All data is reported using pseudonyms.

**Table 2:** Rubric for coding teachers’ NOS views (Irez 2004)

Themes	Naive	Eclectic	Informed
Empirical NOS	Describes science as being solely dependent on direct evidence, believes that scientific claims can (only) be proven by direct evidence.	Believes that science solely relies on direct evidence but accepts that evidence supports rather than proves scientific claims. Believes that science does not solely rely on direct evidence but accepts that evidence proves scientific claims.	Believes science uses both direct and indirect evidence and claims that evidence supports rather than proves scientific claims.
Scientific method	Believes that there is a single universal scientific method which scientists follow step-by-step to reach conclusions.	Believes that there exists a universal scientific method which is not a stepwise procedure.	Believes that there are many methods in science and saw method as related to paradigm.
Tentative NOS	Claims that scientific knowledge is true and certain.	Accepts that some scientific theories are tentative but claims that scientific laws are true and not subject to change.	Believes that all scientific knowledge, regardless to their nature or status, are subject to change and modifications in the future.
Nature of scientific theories and laws	Believes that theories are not well sustained and therefore subject to change. Also claims that, when proven, theories become laws which have higher status and are not subject to change.	Believes in the well-sustained nature of theories, thus views them as subject to change. However, fails to recognize theories and laws as different kinds of scientific knowledge or believes that laws have higher status and are not subject to change.	Believes that theories are well-supported explanation systems. Demonstrates an understanding that theories and laws are different kinds of scientific knowledge, and laws, as well as theories, are subject to change.
Inference and theoretical entities in science	Believes in science’s reliance on direct evidence and therefore does not appreciate the inferential nature of some theories.	Although accepts reliability of some theories which are based on inference, objects to some others claiming that there is no direct evidence to support (or prove) them.	Demonstrates a comprehensive understanding of the inferential nature of some theories.
Subjective and theory-laden NOS	Believes that science pictures an objective account of nature due to its methods and objectivity of its practitioners.	Believes that there could be differences amongst scientists in data interpretation due to their professional backgrounds. Believes that there could be differences amongst scientists in data interpretation due to their personal values and beliefs.	Views subjectivity as integral to the construction of scientific knowledge and believes that scientists professional and personal backgrounds causes subjectivity.
Social and cultural embeddedness of science	Claims that science is universal and denies social and cultural influences on science.	Accepts that society and culture affect some scientific disciplines (such as evolutionary biology), not all (such as chemistry).	Believes that science affects and is affected by society and culture.
Imagination and creativity in science	Rejects that science involves imagination and creativity.	Believes that certain stages of scientific inquiry involve imagination and creativity. On the other hand, holds inconsistent views about scientific methodology and the inferential nature of some scientific theories.	Believes that imagination and creativity permeates the scientific process throughout.

## Results

An improvement was observed in all participant teachers’ NOS views at the end of the innovative NOS-CPD program. Teacher’s naive views about NOS themes decreased whereas their informed views increased (Table 3). On the other hand, it would be expected that the pre-performances of the teachers who had previously taken courses or training about the history of science and philosophy of science or NOS would do better than those without previous experience; but as can be seen from the ratios in the table, there is no significant difference between the pre- and post-performances of the teachers who did and did not take the courses. Before the NOS-CPD program, it was seen that most of the teachers had naive views on the most of the 8-targeted NOS themes, regardless of whether they had taken courses before or not. There is also no significant difference in the increase in the performances of the two groups.

**Table 3:** Personal performances of teachers in the pre- and post-interview. These ratios show how much of the teachers’ views on the eight themes are naive, eclectic, and informed

Teacher	Pre-interview						Post-interview					
	Naive		Eclectic		Informed		Naive		Eclectic		Informed	
	*f*	%	*f*	%	*f*	%	*f*	%	*f*	%	*f*	%
Harun	5	62.5	3	37.5	0	0	0	0	4	50	4	50
*Irmak*	4	50	4	50	0	0	1	12.5	2	25	5	62.5
*Yelda*	5	62.5	3	37.5	0	0	0	0	0	0	4	50
Buse	5	62.5	3	37.5	0	0	1	12.5	5	62.5	2	25
*Zehra*	3	37.5	3	37.5	2	25	0	0	3	37.5	5	62.5
*Lara*	4	50	2	25	2	25	2	25	2	25	4	50
Kerem	5	62.5	2	25	1	12.5	1	12.5	1	12.5	6	75
Akın	4	50	3	37.5	1	12.5	1	12.5	2	25	5	62.5
*Nihan*	2	25	5	62.5	1	12.5	1	12.5	6	75	1	12.5
*Oya*	5	62.5	1	12.5	2	25	1	12.5	3	37.5	4	50
*Mete*	4	50	3	37.5	1	12.5	1	12.5	2	25	5	62.5
Fulya	2	25	2	25	4	50	1	12.5	0	0	8	100
*Gamze*	3	37.5	3	37.5	2	25	1	12.5	1	12.5	6	75
*Sevgi*	1	12.5	4	50	3	37.5	0	0	0	0	8	100
*Sarp*	1	12.5	4	50	3	37.5	0	0	3	37.5	5	62.5
*Can*	1	12.5	2	25	5	62.5	0	0	2	25	6	75
*Efe*	2	25	4	50	2	25	1	12.5	2	25	5	62.5
*Duru*	1	12.5	2	25	5	62.5	0	0	1	12.5	7	87.5

N: 8; in italics are those who had previously taken a course or training about the history of science and philosophy of science or NOS

For the themes specifically targeted by the program, the percent of teachers who had naive views about these themes decreased whereas the percent of teachers with informed increased (Table 4).

**Table 4:** Percentage of teachers’ NOS views in the pre- and post-interview

	Naive		Eclectic		Informed	
	Pre (%)	Post (%)	Pre (%)	Post (%)	Pre (%)	Post (%)
Empirical NOS	55	11	39	34	6	55
Scientific method	72	0	22	34	6	66
Tentative NOS	6	0	78	39	16	61
Nature of scientific theories and laws	83	44	11	22	6	34
Inference and theoretical entities in science	44	6	34	55	22	39
Subjective and theory-laden NOS	22	0	50	39	28	61
Social and cultural embeddedness of science	28	0	6	0	66	100
Imagination and creativity in science	0	0	61	11	39	89

As it is seen on the Table 4, the theme in which the furthest progress was made as a result of the CPD program is “scientific method.” While 72% of the teachers had informed views regarding this theme before the program, the ratio was reduced to zero at the end. All of the teachers comprehended that the scientific method was not the only and universal one.

It is hard to talk about a universal method in general as scientists might have different methods even though they are working on the same subject. (Lara, _post-interview_)

A very considerable increase was observed in the ratio of teachers indicating that the scientific method is not composed of steps that are followed one by one and nor is it unique and universal.

In my opinion, every scientist has his own method. So it is not possible that every scientist follows the same steps in the scientific method. (Irmak, _post-interview_)

The “Imagination and creativity in science” theme is one of those in which a high level of success was achieved in the aftermath of the CPD program. The eclectic level to which 61% of the teachers belonged decreased to 11% after the study and the ratio of informed teachers reached to 89%. Most of the teachers comprehended that scientists use their imagination and creativity at every stage of their studies:

They might use their imagination and creativity at every stage, however they might use it more at some stages. For example, they might use their imagination/creativity less while recording data whereas they use it a lot when making an observation and maybe more when making a deduction. Still, imagination and creativity are present at every stage. (Sevgi, _post-interview_)

As it is seen on the table, the ratio of teachers sharing informed views about “the social and cultural embeddedness of science” theme after the program is 100%. All of the teachers underlined that science was not universal, and scientific studies might be affected from the culture and the values of the society:

The needs of a society, personal needs, religious opinion and even the languages spoken have an effect on scientific studies. (Sarp, _post-interview_)

The improvement achieved in the “nature of scientific theories and laws” theme as a result of the CPD program was less than expected. The percentage of teachers having naive views about the theme before the program decreased from 83 to 44%; however, the percentage of teachers having an informed views about the theme increased from 6 to only 34%. All teachers stressed that the theories might change, but some of them were persistent in their opinion that there was a hierarchical relation between theories and laws and that theories turned into unalterable laws when proved.

I think law is a proved theory. (Akin, _post-interview_)

One of the teachers sharing a conscious opinion after the program while she had naive views regarding the “nature of scientific theories and laws” theme before, clearly underlined her opinion regarding this subject:

I would give a very good answer to that question before; I kept the cliché sentence ‘theories are developed, proven and turn into law’ in my mind for years. However, I do think different now. There might be a mutual interaction. A law might be explained by more than one theory. (Duru, _post-interview_)

## Discussion and conclusion

Our primary research question asked in what ways and to what extent does teachers NOS views change in response to an innovative NOS-CPD program that provided sustained support? In response, our research findings showed that the innovative NOS-CPD program improved teachers’ NOS knowledge and understanding in general. For the themes specifically targeted by the program, the percent of teachers who had naive views about all these themes decreased whereas the percent of teachers with informed increased. As a result of the innovative NOS-CPD program, the NOS theme in which the furthest progress was made is “scientific method.” Another theme in which a high level of progress was made is the “imagination and creativity in science” theme. The improvement achieved in “nature of scientific theories and laws” theme as a result of the innovative NOS-CPD program was less than expected. It is more difficult to ensure improvement in some NOS themes than others even if the direct-reflective teaching method is used. “Nature of scientific theories and laws” is one of those (Koseoglu et al. 2010). The researches carried out in this field claim that the educational (the need for more examples and activities in some subjects than in others), motivational (intrinsic task motivation, performance motivation, utility value, competence belief, self-efficacy, peer support, team work, work a real science research lab.), and socio-cultural (socio-cultural state of the participants, especially with respect to background and possible worldview differences, such as reluctance to accept ambiguity) factors can explain the difficulty of making an improvement in views about this theme (Mesci and Schwartz 2016). It is recommended to be more taken into consideration of these factors mentioned in the literature and to be emphasized in training and activities with extra examples of the NOS themes, which are relatively harder than the other themes.

Our subquestion asked to what extent was our program more effective than short-term professional development programs? In response, our research findings showed that the innovative NOS-CPD program is more successful than short-term programs improving teachers’ views of the NOS themes, especially which are difficult to change as scientific method or the nature of scientific theory and laws. When we looked at the literature, studies have generally demonstrated that short-term professional development programs are difficult to change teachers’ views on such NOS themes (Dogan et al. 2011; Dass and Yager 2009; Torff and Sessions 2008). For example in one study by Dogan et al. (2011) investigating the effects of a 1-week in-service training program on teachers’ views on the nature of science, it is seen that the majority of the views teachers have about NOS have not changed. In this study, it was concluded that such short-term in-service training program was not sufficient in order to be able to make a change in the opinions of teachers, such as theories and laws, where teachers were found to have quite common misconceptions in many studies. As a result of this study, researchers stated, as stated in many studies, many different techniques have to be applied for a longer time to correct such misconceptions. Koseoglu et al. (2011) have also achieved similar results in their experimental works aimed at developing a professional development package for the NOS instruction. One of the important results obtained during the study is that a long process is needed to change the opinions about the NOS. As a result of various activities and debates during the first semester, the rate of good opinions increased to 14.4 and 38.1%, respectively, and it was necessary to apply one more period in order to reach this rate to 67%. In the interim evaluations made, it was seen that most of the participants were aware of the intrinsic insights of the focused science, but inconsistent opinions were seen when their opinions were taken in different contexts. This finding has shown that even a period of education that is explicitly focused on the NOS is not sufficient to internalize the NOS. On the other hand, it has been determined in our study that having already taken a course or training about the history of science and philosophy of science or NOS does not make a difference in the pre- and post-performance of the teachers. The fact that the pre-performances of the teachers who have already taken the courses as they are in the teachers who do not take courses show that the courses they have taken do not have a lasting effect on them. Researches show that short-term professional development programs do not permanently improve teachers’ views about NOS (Akerson and Hanuscin 2007; Koseoglu et al. 2011). Thus, it is thought that this situation may have been caused by the fact that the courses that the teachers had previously attended were short-term.

Learning to teach is a slow process and note easy. Therefore, it should be taken into account that teachers may have difficulty changing their previous knowledge and misconceptions, and professional development programs should be designed for a long time with this prediction (Hayes 1995). In addition, the teachers’ classroom practices should be followed during and after the professional development programs, should be supported to solve the problems encountered in the integration of science with the NOS, and should be provided with the necessary materials in this process (Dogan et al. 2011). It is thought that teacher views about NOS may be improved in this way more permanently and internalized. Researches indicate that teaching the NOS by integrating it to other subjects within the scope of a specific lesson improves teachers’ professional competences about NOS (Schwartz 2009). In addition to providing sustained support, context-specific teaching materials were also provided to teachers in our implemented innovative NOS-CPD program. However, there are limited examples that will guide the teachers in this regard in the literature (Khishfe and Lederman 2003; Schwartz 2009). It is recommended that such exemplifying studies in this area should be increased. Certainly, providing enough time and material is not the only factor that effect CPD program’s effectiveness. Our implemented NOS-CPD program was innovated by including formative assessment and discourse analysis. These innovations are thought to enhance the success of our program. Beside this, as we know from the literature that CPD program’s quality is also affected by other factors (based on teachers’ needs, integration with other reform efforts, high-quality instruction, active engagement, enhancement of both content knowledge and pedagogical content knowledge, improvement of teacher beliefs, continued support, collaboration, reflection and feedback, evaluation procedures, and provision of local support). Therefore, it is recommended that these factors should not be ignored.

The effect of the factors mentioned above in the development of teachers’ NOS views is quite obvious in the literature. However, the main problem for teachers is that they have difficulty integrating what they learn in professional development programs into their classroom practices. It is thought that the use of formative assessment procedures during the teaching process and reinforcement of teaching with discourse analysis and support of teachers through feedback during the process will also improve the performances of the teachers in the classroom. We have also shown that the innovative NOS-CPD program that incorporates these features improved the teachers’ classroom practices. There were other issues that could be reported in this paper, but this paper focused especially on teacher’ NOS views. It is recommended that a professional development program in this context should investigate and report on the effect of teachers, especially on classroom practices.

Last of all, as Guskey (2007) emphasizes, there is a large research base on the professional development of teachers in the literature, but some of these researches are finding opposite findings. For example, while some research suggests that professional development activities should be teacher-specific and focus on daily classroom activities, some researches do not give importance to them and require more holistic and organizational approaches. Some experts state that professional development reforms must be initiated/carried out by teachers or school personnel. Others say they need guidance with a clear vision because they do not have the opportunity to think a wide variety of change and practice. Therefore, the biggest problem in determining the characteristics of successful professional development programs is trying to find a “single correct answer.” General prescriptions cannot provide much guidance to practitioners because “context” is a powerful influence. In one context, there may be a need for a managerial structure in another context, while teacher-led activities are in need. In other words, instead of “one correct answer” or “one correct path,” there is a collection of answers developed according to context. So the aim should be to find the most suitable mixture and to be aware that this mixture may change over time (Guskey 2007, as cited in Bumen et al. 2012). Based on our literature searches and implementation, we may say that our innovative NOS-CPD program contains the one of the most suitable mixture for developing teachers’ professional competences about NOS. But of course, this program may be improved by experimenting in different contexts.

## Abbreviations

CPD: Continuing Professional Development
MONE Turkey: Ministry of National Education of Turkey
NGSS Lead States: Next Generation Science Standards in the United States
NOS: Nature of Science
VNOS-C: Views of Nature of Science-Form C
